# Parliament and the Professions

**Published:** 1920-07-24

**Authors:** 


### Parliament and the Professions.

### Third London General Hospital

THE Minister of Pensions informed Mr. Macpherson that the Third London General Hospital had been offered to the Ministry; but the accommodation was considered less suitable for the requirements of the Ministry than that of another hospital, the transfer of which to the Ministry is being considered. The number of war pensioners now receiving treatment in civil hospitals is approximately 15,000.

### Cost of National Health Insurance.

DR. ADDISON stated that the total cost of National Health Insurance from its inception to the end of 1919 was, in round figures, £190,000,000; the amounts paid in benefits and administration were £99,000,000 and £25,000,000 respectively, leaving a balance in reserve of £66,000,000.

### Financial Position of Hospitals

IN answer to a question put by Major Godfrey Palmer, Dr. Addison stated that he had for some time past been giving the most anxious consideration to the future of voluntary hospitals. The situation in London has been relieved by the recent emergency distribution of £250,000 by King Edward's Hospital Fund, and the position of the provincial hospitals is generally less serious than in London. The question of State assistance raises many difficult issues, and he is anxious to avoid any precipitate measures which might prejudice the voluntary principle to the maintenance of which he attaches the greatest importance.

The Minister made a further allusion to this matter during the debate on Supply, which is referred to under "Hospital and Institutional News."

